# Estimation of prevalence and complications of iron deficiency anemia in pregnancy at Obio-Akpor, Rivers State, Nigeria from 2019 to 2023

**DOI:** 10.1097/MD.0000000000047666

**Published:** 2026-02-13

**Authors:** Getrude Uzoma Obeagu, Basil Omieibi Altraide, Emmanuel Ifeanyi Obeagu

**Affiliations:** a Department of Midwifery and Child health Nursing, University of PortHarcout, Rivers State, Nigeria; b Department of Obstetrics and Gynaecology, Rivers State University Teaching Hospital, Port Harcourt; c Department of Biomedical and Laboratory Science, Africa University, Mutare, Zimbabwe.

**Keywords:** complications, iron deficiency anemia, pregnancy, prevalence, red cell indices

## Abstract

Iron deficiency anemia (IDA) is the most common form of anemia in pregnancy and remains a significant public health problem worldwide, particularly in low- and middle-income countries. It is one of most common nutritional disorders associated with adverse maternal and fetal complications and increased perinatal mortality. This study aimed to determine the prevalence of IDA among pregnant women and maternal and fetal complications at Churchill and Rumunduru Model Health Centers in Obio-Akpor, Rivers State, Nigeria, between 2019 and 2023. A retrospective descriptive cross-sectional design was employed. The study reviewed 2290 antenatal records of pregnant women, 458 yearly and 229 per health center annually. Structured data extraction form was used to collect information on socio-demographic characteristics, obstetric history, hemoglobin levels, mean corpuscular volume, maternal and fetal complications. Systematic random sampling was applied to select eligible records. Prevalence of IDA in pregnancy revealed values of 58.60%, 74.00%, 60.10%, 54.10%, and 63.10% for 2019, 2020, 2021, 2022, and 2023, respectively. Prevalence values ranged between 54.1% and 74.0% while overall value was 62.0%. Maternal complications observed included pre-eclampsia (9.1–22.3%), post-partum hemorrhage (10.5–25.1%), pre-term labor (7.4–22.3%), maternal distress (14.8–27%), pre-term birth (1.3–6.4%), pre-natal infection (0.4%), and post-natal infection (1.8–7.4%). Fetal complications identified were low birth weight (5.3–26.6%), pre-term (11.9–48%), fetal distress (13.2–21.8%), intrauterine growth restriction (0.4–5.5%), and fetal death (2.2–6.1%). IDA remains highly prevalent among pregnant women in Obio-Akpor, Rivers State, and is associated with maternal and fetal complications. The findings underscore the need for strengthened antenatal care interventions, including early screening, iron supplementation, nutritional counseling, and close monitoring of at-risk pregnancies to reduce adverse outcomes.

## 1. Introduction

Iron deficiency anemia (IDA) in pregnancy is one of the most important global health challenges, particularly in low- and middle-income countries where nutritional deficiencies, infections, and poverty interact to increase vulnerability. IDA was defined by American College of Obstetricians and Gynecologists as a hemoglobin (Hb) value <11.0 g/dL as well as mean corpuscular volume (MCV) lower than normal range of 80 fl to 100 fl or serum ferritin level <30 ng/mL.^[1]^ World Health Organization (WHO)^[2]^ estimated that 50% of the anemia seen in pregnancy is due to iron deficiency, defined anemia in pregnancy as an ill health with Hb concentration <11 g/dL and further classified it with a Hb values of 9 to 10.9 g/dL as mild anemia, 7 to 8.9 g/dL as moderate anemia and lower than 7 g/dL as severe anemia. WHO reported that 37% of pregnant women worldwide are anemic,^[3]^ and the condition contributes substantially to maternal and perinatal morbidity and mortality.

Pregnancy is a period of increased physiological demand.^[4]^ It begins with fertilization, a process of fusion of male and female gametes to form a zygote which passed developmental stage of embryo to fetus.^[5]^ Maternal blood volume expands by almost 50%, leading to higher requirements for iron to support erythropoiesis, fetal growth, and placental development.^[4]^ When these demands are not adequately met, either due to low dietary intake, poor absorption, or chronic blood loss, iron deficiency develops. IDA is the most common type of anemia in pregnancy apart from hemodilution. Iron deficiency is usually caused by inadequate dietary iron intake, is considered the most common nutritional deficiency leading to anemia.^[6]^ Deficiencies of vitamin A, folate, vitamin B12, and riboflavin can also cause anemia due to their specific roles in the synthesis of Hb and/or erythrocyte production.^[2]^

IDA is highly prevalent in pregnancy and represents a major cause of adverse maternal and fetal outcomes.^[6]^ The consequences of IDA are far-reaching. For the mother, it increases the risk of fatigue, reduced work capacity, post-partum hemorrhage, sepsis, heart failure, and in severe cases, maternal death. For the fetus and neonate, IDA is associated with pre-term birth, intrauterine growth restriction, low birth weight, stillbirth, impaired psychomotor development, and increased risk of perinatal mortality.^[7]^ Thus, the burden of IDA extends beyond pregnancy and has long-term implications for child survival and development.^[8]^ The burden is most severe in sub-Saharan Africa, where poor nutritional intake, endemic malaria, parasitic infections, and limited access to healthcare services converge to increase the risk of IDA in pregnancy.^[8]^

Globally, in 2019, 36.5% in pregnant women^[9]^ and 37% (32 million) of pregnant women were affected by anemia.^[2]^ 52% in developing countries compared with 23% in developed countries.^[9]^ In India, IDA prevalence was 50% to 70%.^[10]^ Australia and United States of America, it ranged between 18% and 20%.^[11,12]^ In Nigeria, IDA remains one of the leading medical disorders in pregnancy. IDA prevalence in Pregnancy were 25% to 46% in Lagos and Kano, North West based on Hb value and serum ferritin.^[13]^ Nwanguma et al,^[14]^ in Enugu, South East reported 23.6% IDA prevalence among pregnant woman based on Hb value and serum ferritin. In South-South, hospital-based studies report prevalence rates ranging between 32% and 76%, using laboratory values of Hb and serum ferritin with variations across geographic and socio-economic groups in Cross River.^[15]^ Obeagu et al,^[16]^ reported 61% in Akwa Ibom using Hb value while in Rivers State, Ringshaw et al,^[17]^ reported 60% prevalence of IDA in pregnancy using laboratory values of Hb and serum ferritin.

Contributing factors to high rate of IDA occurrences include poor nutrition, high rates of malaria and helminth infestations, late booking for antenatal care, noncompliance with iron supplementation, poverty, and cultural food taboos that limit iron-rich diets.^[18]^ WHO recommends daily iron of 30 to 60 mg and folic acid 400 µg (0.4 mg) supplementation in pregnant women throughout pregnancy beginning as early as possible for pregnant adolescents and adult women in all health care settings.^[19]^ Also, in place where anemia in pregnancy is a severe public health problem, a daily dose of 60 mg of elemental iron is recommended. In addition, when a pregnant woman is diagnosed with anemia in hospital, daily iron 120 mg and folic acid 400 µg or 0.4 mg supplementation should be given until her Hb level rises to normal and continued with the standard antenatal dose to prevent recurrence of anemia.^[20]^ Ideally, iron and folic acid supplementation should form part of an integrated program of antenatal and neonatal care that promotes healthy pregnancy outcome.^[9]^ Despite the availability of antenatal interventions such as iron and folic acid supplementation, intermittent preventive treatment for malaria, and nutritional health education, the prevalence of IDA remains unacceptably high in many parts of developing country like Rivers State, Nigeria.

Rivers State, located in the oil-rich Niger Delta region of Nigeria, is characterized by rapid urbanization, socio-economicinequalities, and diverse health-seeking behaviors.^[18]^ Obio-Akpor, one of the most populous and urbanized Local Government Area (LGA) in the state, has 2 model health facilities offering antenatal services. However, anecdotal reports and clinical observations suggest that anemia in pregnancy remains a common finding among women receiving care in these facilities. The socio-economic and cultural diversity in Obio-Akpor, coupled with challenges of healthcare access, irregular antenatal attendance, and dietary practices, may contribute to the persistence of IDA in pregnancy in this setting.^[21]^ The period between 2019 and 2023 has been particularly critical for maternal healthcare delivery in Nigeria. The COVID-19 pandemic, which peaked during this time, disrupted access to health services, including antenatal care, while economic hardships limited household food security and dietary quality.^[18]^ These contextual challenges may have influenced the burden of anemia in pregnancy, yet there is limited empirical data documenting the prevalence and outcomes during this period in Obio-Akpor.

Understanding the prevalence and complications of IDA in pregnancy within Obio-Akpor, Rivers State, Nigeria is crucial for several reasons. First, it provides local epidemiological evidence necessary for designing targeted interventions to reduce maternal and neonatal morbidity and mortality. Second, it will inform policymakers and healthcare providers about the effectiveness of existing antenatal strategies and identify gaps in service delivery. Third, documenting complications associated with IDA such as post-partum hemorrhage, need for blood transfusion, pre-term birth, and low birth weight will highlight the public health significance of addressing this preventable condition. This present study seeks to estimate the prevalence and complications of iron deficiency anemia (IDA) in pregnancy at Obio-Akpor, Rivers State, Nigeria, between 2019 and 2023.

## 2. Materials and methods

### 2.1. Research design

The study adopted retrospective descriptive cross-sectional design. The design is considered appropriate because the study determined the prevalence and complications of IDA in pregnancy using existing antenatal records between 2019 and 2023. In this study, prevalence of IDA was the proportion of pregnant women with Hb value <11 g/dl and MCV lower than normal range of 80 fl to 100 fl expressed in percentages. Serum ferritin was not used in this study because it was not documented as part of laboratory investigations on antenatal records. The study was done in a community hospital in a low resource setting that lacks resources to offer laboratory iron investigations to pregnant women. The adverse pregnancy outcomes among respondents that had IDA were referred to as maternal and fetal complications of IDA presented in percentages. The antenatal records were carefully reviewed to exclude records of pregnant women with chronic diseases, kidney disease, sickle cell disorders, and thalassemia. A retrospective approach allows for the review and analysis of documented data on pregnant women who received antenatal care and delivered in Churchill Model Health Centre (CMHC) and Rumunduru Model Health Centre (RMHC) in Obio-Akpor, Rivers State, Nigeria. By examining antenatal records, antenatal case notes, laboratory reports, and delivery report, the study generated information on the proportion of pregnant women who were diagnosed with IDA as well as the associated maternal and fetal complications documented during the study period. The cross-sectional nature of the design enables the researcher to capture and describe the burden of IDA in pregnancy at a specific period of time (2019–2023) without manipulating any variables. This design is also cost-effectiveand time-efficient, as it utilizes already available secondary data. The descriptive aspect of the design provides a quantitative summary of the prevalence and complications of IDA.

### 2.2. Study area

This study was conducted at CMHC and RMHC, both located in Obio-Akpor, Rivers State, Nigeria. Obio-Akpor is one of the 23 LGA of Rivers State and is part of the metropolitan area of Port Harcourt, the state capital. It is highly urbanized and densely populated, with residents engaged in business, civil service, oil and gas employment, and various informal economic activities. The LGA has a high demand for maternal and child health services due to its large youthful population and relatively high fertility rate.

CMHC is a government-owned primary healthcare facility located within Obio-Akpor. It offers a range of maternal and child health services including antenatal care (ANC), routine laboratory investigations, immunization, delivery services, and family planning. The facility serves a large catchment population drawn from Churchill community and surrounding areas. Pregnant women attending ANC at the center undergo routine Hb screening, enabling early detection and management of anemia.

RMHC is another public primary healthcare facility situated in Rumunduru community, Obio-Akpor. Like CMHC, it provides essential reproductive, maternal, newborn, and child health (RMNCH) services. The facility records antenatal attendance from women within Rumunduru and neighboring communities. Standard ANC services include Hb estimation, health education, nutritional counseling, iron and folate supplementation, and skilled delivery care.

Both CMHC and RMHC were purposively selected because they are major government facilities within Obio-Akpor that maintain reliable antenatal and delivery registers, making them suitable for retrospective review of cases between 2019 and 2023. The 2 centers provide a representative picture of the burden of IDA in pregnancy and related maternal and fetal complications within the LGA.

## 2.3. Study population

The study population consist of pregnant women who attended ANC clinics and/or delivered in CMHC and RMHC within Obio-Akpor, Rivers State, Nigeria between January 2019 and December 2023. The population of interest specifically includes women of reproductive age (15–49 years) who became pregnant during the study period and accessed maternal health services in the health centers. These women are routinely screened for anemia during ANC through Hb estimation or full blood count, and their records often capture socio-demographic details, gestational age at booking and subsequent visits, obstetric history, laboratory results, and delivery outcomes. This population is appropriate for the study because it reflects the burden of IDA in pregnancy in the locality and provides the opportunity to assess associated maternal and fetal complications within the 5-year period under review.

### 2.3.1. Inclusion criteria. 

Pregnant women who received antenatal care at the selected health centers from 2019 to 2023. Women with recorded Hb or other relevant laboratory results indicating IDA. Availability of complete demographic data and clinical information in the antenatal records.

### 2.3.2. Exclusion criteria. 

Pregnant women with known chronic medical conditions (e.g., chronic diseases, kidney disease, sickle cell disease, thalassemia) that may complicate IDA. Incomplete antenatal records or missing key information example, no Hb levels recorded, women who only attended antenatal care sporadically without complete follow-up data.

### 2.3.3. Study sample. 

The sample for this study consists of 2290 records of pregnant women for the 5 years period, 458 records each year, and 229 antenatal records of pregnant women per health center per year, who attended ANC at CMHC and RMHC in Obio-Akpor, Rivers State from 2019 to 2023. The sampling frame was the list of all pregnant women who have attended ANC in the health centers during the study period. This list was obtained from antenatal care registers.

### 2.3.4. Sample size calculation. 

The sample size was determined using the formula for estimating proportions in a population based on the estimated prevalence of IDA, desired precision, and confidence level:

Sample size calculation formula:

$n=\frac{z^{2}pq}{d^{2}}$ *q* = 1 − *p. d* = degree of accuracy set at 0.05.

where, *n* = the desired sample size.

*z* = the standard normal deviate, usually set at 1.96 which corresponds to 95% confidence level.

*p* = the estimated percentage or prevalence of the attribute that is present in the population that is IDA in pregnancy as Obeagu et al^[16]^ reported 61.1% (0.61) prevalence of anemia in pregnancy).

*n* = 1.96 × 1.96 × 0.61 × 1 − 0.61 ÷ 0.05 × 0.05 = 3.8416 × 0.61 × 0.39 ÷ 0.0025 = 0.91391664 ÷ 0.0025 = 457. Each health center had a sample size of 229 (457 ÷ 2). The researcher adopted same sample size based on expected prevalence for each year.

### 2.3.5. Sampling techniques. 

This study adopted the Systematic Random Sampling technique to select records of pregnant women from the antenatal and delivery registers of CMHC and RMHC in Obio-Akpor. Systematic random sampling is a probability sampling method in which every kth element in the sampling frame is selected after a random starting point. This technique was chosen because the study involves retrospective data from antenatal care registers that are often arranged in chronological order of attendance, making it easier and more practical to apply.

### 2.3.6. Methods of data collection. 

The study used secondary data obtained from antenatal clinic care registers from the selected rural hospitals, which include Hb levels, MCV, patient demographic data, clinical history, and pregnancy complications in Obio-Akpor, Rivers State, Nigeria between January 2019 and December 2023. The primary sources of data include:

### 2.3.7. Antenatal registers. 

These contain information on pregnant women’s socio-demographic characteristics (age, marital status, and occupation), obstetric history (gravidity, parity, and gestational age), medical history, Hb results, red blood cell indices, maternal and fetal complications among others.

### 2.3.8. Laboratory reports. 

These provide Hb concentrations, red blood cell indices specifically MCV conducted during pregnancy.

### 2.3.9. Delivery registers and case notes. 

These provide maternal and fetal complications such as mode of delivery, post-partum hemorrhage, maternal transfusions, maternal death, birth weight, Apgar score, pre-term delivery, stillbirth among others. Detailed patient notes was used to confirm Hb levels, diagnosis of IDA, interventions, and complications.

### 2.3.10. Data collection tool. 

A structured data extraction form was developed to systematically collect the relevant information from the antenatal records. The form was divided into sections capturing:

• Socio-demographic characteristics: name (anonymized), hospital number and age.
• Obstetric history: gravidity, parity, gestational age.
• Laboratory results: hemoglobin concentration, CBC specifically MCV.
• Maternal complications: post-partum hemorrhage, sepsis, blood transfusion, maternal distress, maternal death.
• Fetal complications: low birth weight, pre-term birth, stillbirth, among others.
• Other clinical information: medical conditions like chronic diseases, kidney disease, sickle cell disease, thalassemia, malaria in pregnancy and comorbidities.

Pretesting and validation of the instrument: the data extraction form was pretested using 10% of ANC records from the selected health centers that are not included in the main study. Pretesting ensures that the form is clear, comprehensive, and capable of capturing all relevant information. Necessary adjustments was made based on the pretest, such as rephrasing unclear variables or adding missing variables.

## 2.4. Data collection procedure

• Permission and access: ethical approval was obtained from University of Port Harcourt Research Ethics Committee (UNIPORT REC). Permission was gotten from management of CMHC and RMHC in Obio-Akpor, Rivers State, Nigeria to access antenatal and delivery records.
• Recruitment of data collectors: 2 trained research assistants (a midwife and a hematologist in medical laboratory science) were recruited and trained on the use of the data extraction form, confidentiality, and accurate data recording.
• Identification of eligible records: the total population of pregnant women who attended ANC or delivered between 2019 and 2023 was enumerated from the ANC registers. Eligible records were identified based on the inclusion and exclusion criteria.
• Systematic random sampling: using the calculated sampling interval, records was selected systematically to ensure representativeness. The 1st record was randomly chosen, and subsequent records selected at every *k*th interval.
• Extraction of data: the trained research assistants extracted the relevant information from the selected records and enter it into the structured data extraction form. The principal investigator cross-checked a sample of the forms for accuracy and completeness.
• Anonymization: to maintain confidentiality, personal identifiers such as names, hospital numbers, and addresses was removed, and unique study codes assigned to each record.
• Data handling and storage: extracted data were entered into a secure computer spreadsheet (Microsoft Excel). Access was limited to the research team. Backup copies was maintained to prevent data loss.

### 2.5. Methods of data analysis

: the data analysis methods for this study was descriptive statistics such as frequency and percentage to calculate the rate of recurrence and proportions of pregnant women with IDA and identified pregnancy complications. Inferential statistics like chi-square was used to show the association between prevalence rate of IDA in pregnancy across different years as well as health centers and associated pregnancy complications using SPSS version 27 (IBM Corp. IBM SPSS Statistics for Windows, Armonk). Trend analysis help to analyze trends in the prevalence of IDA over the study period. Data was presented on strata by health centers and year focused using graphs and tables.

### 2.6. Ethical considerations

Ethical approval was obtained from UNIPORT REC with REC No: UPH/CEREMAD/REC/MM106/011. The study was conducted in a manner that respects the rights, dignity, and well-being of the participants by ensuring that respondents’ data were kept confidential and anonymized to protect personal information.

## 3. Results

### 3.1. Prevalence of IDA in pregnancy

**Figure 1.**
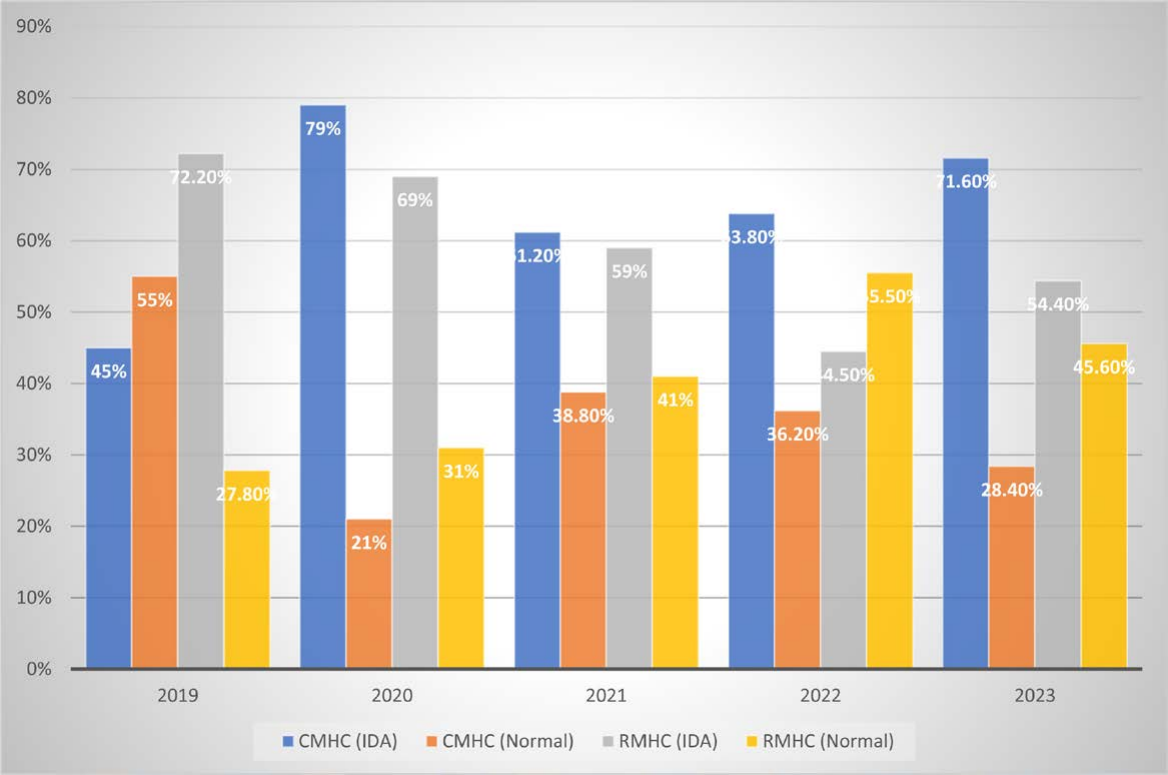
Prevalence of iron deficiency anemia in pregnancy among the respondents stratified by healthcare centers and year of focus.

Figure 1 showed the prevalence of IDA among the respondents stratified by health centers and year focused. Fifty-five percent of the respondents at CMHC in 2019 were normal, while 45% had IDA. In RMHC, 72.2% had IDA while 27.8% had no IDA. In year 2020 at CMHC, 79.0% had IDA, while 21.0% were normal. At RMHC 69% had IDA, and 31.0% were free. Amongst those who registered at CMHC in 2021, 61.2% had IDA, and 38.8% were normal. At RMHC, 59.0% had IDA and 41.0% had no IDA. Majority 63.8% of the pregnant women who registered at CMHC had IDA, while 36.2% were normal. In this same year, 55.5% of those who registered at RMHC had no IDA while 44.5% had IDA. About 71.6% at CMHC in 2023 had IDA, and 28.4% were normal while, 54.4% at RMHC in this same year had IDA and 45.6% had no IDA.

**Figure 2.**
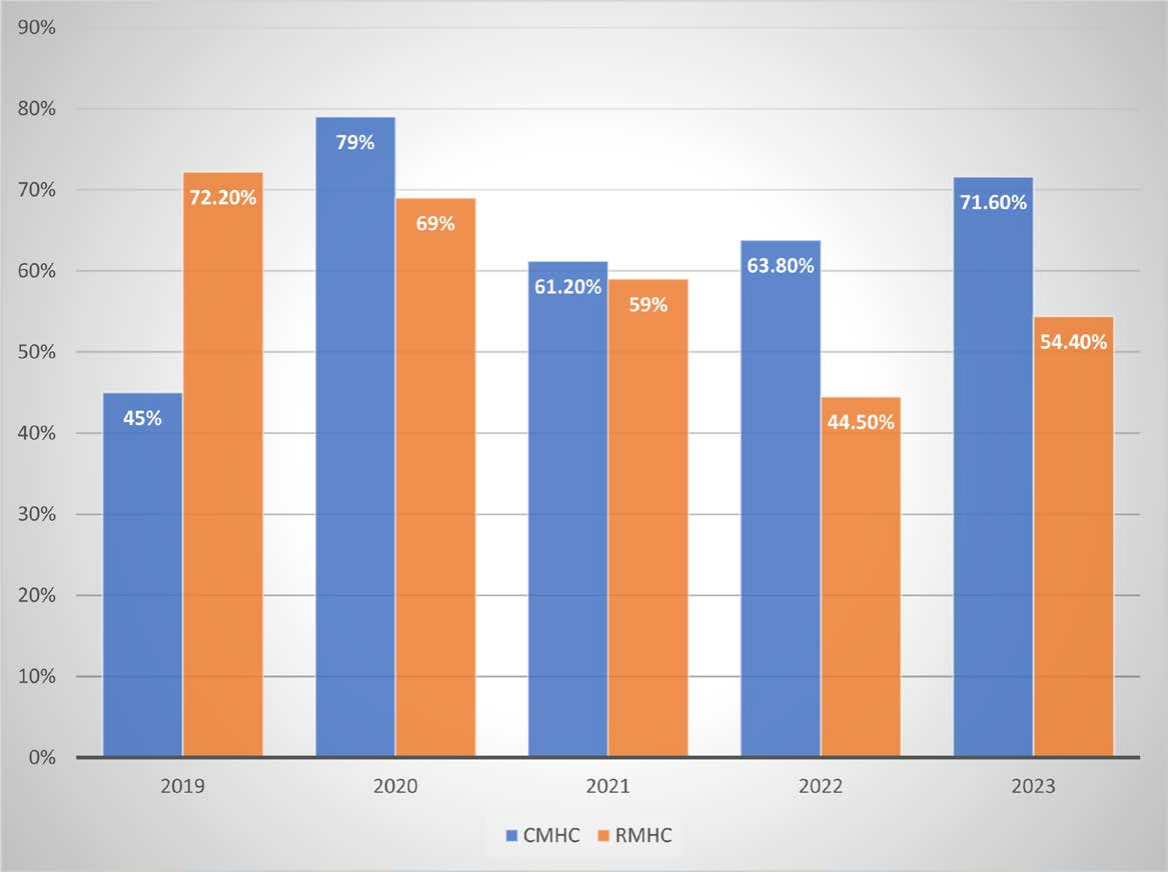
Prevalence of iron deficiency anemia in pregnancy stratified by healthcare centers and year of focus.

Figure 2 showed the prevalence of IDA, stratified by health centers and year focused. In CMHC, IDA prevalence was 45%, 79%, 61.2%, 63.8%, and 71.6% while in RMHC, IDA values were 72.2%, 69%, 59%, 44.5%, and 54.4% for 2019, 2020, 2021, 2022, and 2023 respectively.

**Figure 3.**
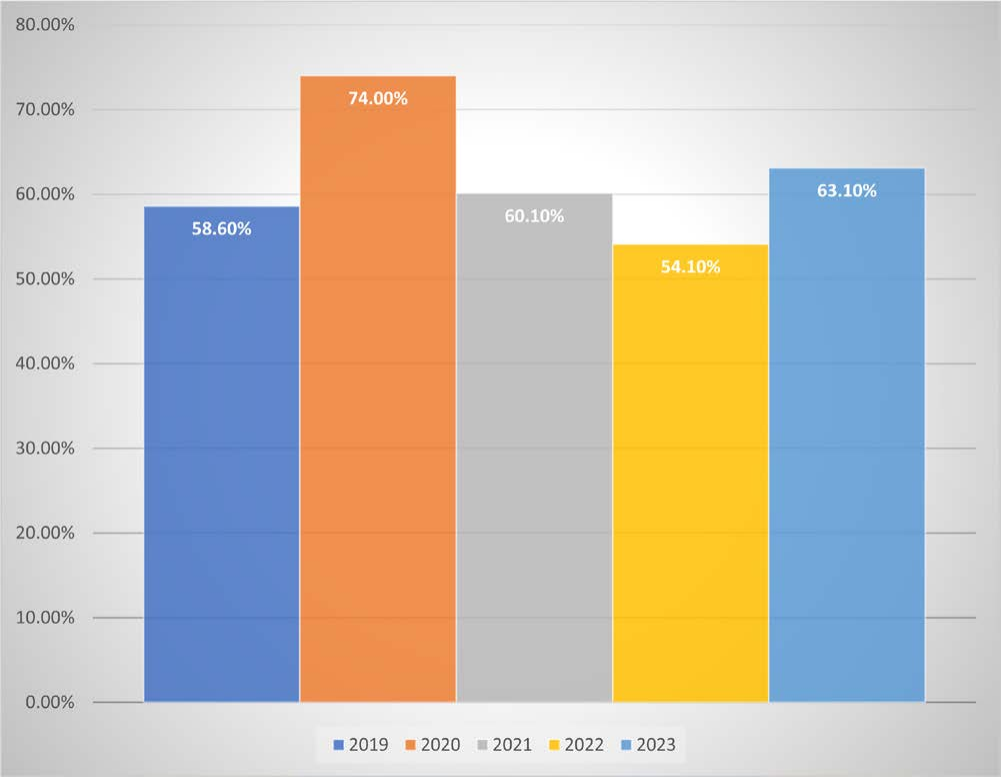
Prevalence of iron deficiency anemia in pregnancy stratified by year focus from 2019 to 2023.

Figure 3 reported the yearly prevalence of IDA in pregnancy with values of 58.60%, 74.00%, 60.10%, 54.10%, and 63.10% for 2019, 2020, 2021, 2022, and 2023, respectively to reveal the trends in IDA prevalence over the study period. Yearly IDA prevalence values ranged between 54.1% and 74.0% while the total prevalence across 5 years was 62.0%.

### 3.2. Maternal complications observed among the respondents

Tables 1 and 2 show the maternal complications observed among the respondents. In 2019, 11.4% experienced pre-term labor at CMHC, while 12.8% experienced pre-term labor at RMHC. At CMHC 14.4% experienced pre-eclampsia,10.5% had post-partum hemorrhage (PPH) while 14.8% and 1.3% had maternal distress and pre-term birth, respectively. In this same year at RMHC, 11.0% and 11.9% of the respondents experienced pre-eclampsia and PPH. 0.4% was observed to have pre-natal infection, while 4.0% and 18.1% had a post-natal infection and maternal distress, respectively. In 2020 at CMHC 1.8% experienced post-natal infection, pre-term birth, and PPH, respectively. About 9.1% experienced pre-eclampsia, and 11.9% had PPH. Twenty-seven percent at CMHC had maternal distress, and 6.4% had pre-term birth. At RMHC, 20.5% of respondents had pre-term labor, 11.4%, 21.8% of respondents had PPH and maternal distress, respectively. At Churchill in 2021, 20.7%, 25.1%, and 3.1% had pre-eclampsia, PPH, and pre-term birth, respectively. In RMHC, 13.1% experienced pre-term labor. About 16.6%, 17.5%, 10.0%, and 1.7% had pre-eclampsia,PPH, maternal distress, and pre-term birth, respectively. In 2022, at CMHC 22.3% and 18.8% had pre-eclampsia and PPH, while in RMHC, 14.0% had pre-term labor. About 14.8%, 17.0%, 10.9%, and 0.4% experienced pre-eclampsia, PPH, maternal distress, and pre-term birth, respectively. At CMHC in 2023, 9.2% had pre-term labor, 18.8% had pre-eclampsia, 18.3% had PPH, 7.4% had maternal distress 1.3% had pre-term birth, 7.4% had post-natal infection, and 0.4% had pre-natal infection.

**Table 1:** Maternal complications observed among the respondents stratified by year of focus and healthcare center.

	2019		2020		2021		2022		2023	
	Churchill MHC	Rumunduru MHC	Churchill MHC	Rumunduru MHC	Churchill MHC	Rumunduru MHC	Churchill MHC	Rumunduru MHC	Churchill MHC	Rumunduru MHC
Maternal complications	*F* (%)	*F* (%)	*F* (%)	*F* (%)	*F* (%)	*F* (%)	*F* (%)	*F* (%)	*F* (%)	*F* (%)
Pre-term labor	26 (11.4)	29 (12.8)	0 (0.0)	47 (20.5)	0 (0.0)	30 (13.1)	0 (0.0)	32 (14.0)	21 (9.2)	17 (7.5)
No complication	109 (47.6)	95 (41.9)	114 (52.1)	106 (46.3)	116 (51.1)	94 (41.0)	135 (59.0)	98 (42.8)	85 (37.1)	89 (39.0)
Pre-eclampsia	33 (14.4)	25 (11.0)	20 (9.1)	0 (0.0)	47 (20.7)	38 (16.6)	51 (22.3)	34 (14.8)	43 (18.8)	37 (16.2)
Post-partum hemorrhage	24 (10.5)	27 (11.9)	26 (11.9)	26 (11.4)	57 (25.1)	40 (17.5)	43 (18.8)	39 (17.0)	42 (18.3)	35 (15.4)
Maternal distress	34 (14.8)	41 (18.1)	6 (2.7)	50 (21.8)	0 (0.0)	23 (10.0)	0 (0.0)	25 (10.9)	17 (7.4)	47 (20.6)
Pre-term birth	3 (1.3)	0 (0.0)	14 (6.4)	0 (0.0)	7 (3.1)	4 (1.7)	0 (0.0)	1 (0.4)	3 (1.3)	3 (1.3)
Post-natal infection	0 (0.0)	9 (4.0)	4 (1.8)	0 (0.0)	0 (0.0)	0 (0.0)	0 (0.0)	0 (0.0)	17 (7.4)	0 (0.0)
Pre-natal infection	0 (0.0)	1 (0.4)	0 (0.0)	0 (0.0)	0 (0.0)	0 (0.0)	0 (0.0)	0 (0.0)	1 (0.4)	0 (0.0)
Pre-term birth & PPH	0 (0.0)	0 (0.0)	4 (1.8)	0 (0.0)	0 (0.0)	0 (0.0)	0 (0.0)	0 (0.0)	0 (0.0)	0 (0.0)
PPH & maternal distress	0 (0.0)	0 (0.0)	8 (3.7)	0 (0.0)	0 (0.0)	0 (0.0)	0 (0.0)	0 (0.0)	0 (0.0)	0 (0.0)

MHC = Model Health Centre, PPH = post-partum hemorrhage.

**Table 2:** Maternal complications observed among the respondents stratified by year of focus and healthcare center.

	2019		2020		2021		2022		2023	
	Churchill MHC	Rumunduru MHC	Churchill MHC	Rumunduru MHC	Churchill MHC	Churchill MHC	Rumunduru MHC	Churchill MHC	Rumunduru MHC	Churchill MHC
Maternal complications	*F* (%)	*F* (%)	*F* (%)	*F* (%)	*F* (%)	*F* (%)	*F* (%)	*F* (%)	*F* (%)	*F* (%)
Pre-term birth & maternal distress	0 (0.0)	0 (0.0)	4 (1.8)	0 (0.0)	0 (0.0)	0 (0.0)	0 (0.0)	0 (0.0)	0 (0.0)	0 (0.0)
Pre-eclampsia & pre-term birth	0 (0.0)	0 (0.0)	1 (0.5)	0 (0.0)	0 (0.0)	0 (0.0)	0 (0.0)	0 (0.0)	0 (0.0)	0 (0.0)
Pre-term birth & post-natal infection	0 (0.0)	0 (0.0)	1 (0.5)	0 (0.0)	0 (0.0)	0 (0.0)	0 (0.0)	0 (0.0)	0 (0.0)	0 (0.0)
Pre-eclampsia & post-natal infection	0 (0.0)	0 (0.0)	4 (1.8)	0 (0.0)	0 (0.0)	0 (0.0)	0 (0.0)	0 (0.0)	0 (0.0)	0 (0.0)
Pre-eclampsia & PPH	0 (0.0)	0 (0.0)	5 (2.3)	0 (0.0)	0 (0.0)	0 (0.0)	0 (0.0)	0 (0.0)	0 (0.0)	0 (0.0)
Pre-eclampsia & maternal distress	0 (0.0)	0 (0.0)	5 (2.3)	0 (0.0)	0 (0.0)	0 (0.0)	0 (0.0)	0 (0.0)	0 (0.0)	0 (0.0)
Post-natal infection & maternal distress	0 (0.0)	0 (0.0)	1 (0.5)	0 (0.0)	0 (0.0)	0 (0.0)	0 (0.0)	0 (0.0)	0 (0.0)	0 (0.0)
PPH & post-natal infection	0 (0.0)	0 (0.0)	2 (0.9)	0 (0.0)	0 (0.0)	0 (0.0)	0 (0.0)	0 (0.0)	0 (0.0)	0 (0.0)

MHC = Model Health Centre, PPH = post-partum hemorrhage.

### 3.3. Fetal complications observed among the respondents

Table 3 shows the fetal complications observed among the respondents. In 2019, at CMHC 17.9% had low birth weight, 24.5% had pre-term birth, and 21.8% had fetal distress. In RMHC, 33.5% of the respondents had pre-term birth, 19.4% had low birth weight, and 2.2% had fetal death. Amongst the respondents at CMHC in 2020, 11.9%, 13.2%, 3.7%, 5.5%, 2.3%, and 1.8% had low birth weight, pre-term birth, fetal distress, fetal death, intrauterine growth restriction, intrauterine growth restriction, and fetal distress, respectively. In the same year at RMHC, 5.3% had low birth weight, 31.0% had pre-term birth, 17.9% had fetal distress, and 1.7% had intrauterine growth restriction. In 2021, at CMHC, 35.7% had pre-term birth, 16.7% had low birth weight, 15.0% had fetal distress, and 0.4% had intrauterine growth restriction. In RMHC, 38.9% had pre-term birth, 21.8% had low birth weight, 10.0% had fetal distress, and 3.5% had intrauterine growth restriction. Among those who registered at CMHC in the year 2022, 39.3% pre-term births, 16.6% had low birth weight, and 14.4% had fetal distress. However, those who registered at RMHC in this same year 48% had pre-term birth and 23.6% had low birth weight, 10% of fetal distress, 2.6% of fetal death, and 0.9% of intrauterine growth restriction. In 2023, 26.6% of those who registered at CMHC had a low birth-weight baby, while 18.0% of those who registered at RMHC had the same child birth outcome. About 34.1% in CMHC and 25.9% in RMHC had pre-term birth. Fetal distress was reported in the CMHC and RMHC with 18.3% and 18.9%, respectively. Fetal death was reported with a percentage of 3.1% and 6.1% in CMHC and RMHC, respectively.

**Table 3:** Fetal complications observed among the respondents stratified by year of focus and healthcare center.

	2019		2020		2021		2022		2023	
	Churchill MHC	Rumunduru MHC	Churchill MHC	Rumunduru MHC	Churchill MHC	Rumunduru MHC	Churchill MHC	Rumunduru MHC	Churchill MHC	Rumunduru MHC
Fetal complications	*F* (%)	*F* (%)	*F* (%)	*F* (%)	*F* (%)	*F* (%)	*F* (%)	*F* (%)	*F* (%)	*F* (%)
Low birth weight	41 (17.9)	44 (19.4)	26 (11.9)	35 (15.3)	38 (16.7)	50 (21.8)	38 (16.6)	54 (23.6)	61 (26.6)	41 (18.0)
No fetal complications	82 (35.8)	65 (28.6)	101 (46.1)	78 (34.1)	73 (32.2)	59 (25.8)	68 (29.7)	34 (14.8)	41 (17.9)	71 (31.1)
Pre-term birth	56 (24.5)	76 (33.5)	26 (11.9)	71 (31.0)	81 (35.7)	89 (38.9)	90 (39.3)	110 (48.0)	78 (34.1)	59 (25.9)
Fetal distress	50 (21.8)	37 (16.3)	29 (13.2)	41 (17.9	34 (15.0)	23 (10.0)	33 (14.4)	23 (10.0)	42 (18.3)	43 (18.9)
Fetal death	0 (0.0)	5 (2.2)	8 (3.7)	0 (0.0)	0 (0.0)	0 (0.0)	0 (0.0)	6 (2.6)	7 (3.1)	14 (6.1)
Intrauterine growth restriction	0 (0.0)	0 (0.0)	12 (5.5)	4 (1.7)	1 (0.4)	8 (3.5)	0 (0.0)	2 (0.9)	0 (0.0)	0 (0.0)
Intrauterine growth restriction and fetal distress	0 (0.0)	0 (0.0)	5 (2.3)	0 (0.0)	0 (0.0)	0 (0.0)	0 (0.0)	0 (0.0)	0 (0.0)	0 (0.0)
Fetal distress & low birth weight	0 (0.0)	0 (0.0)	4 (1.8)	0 (0.0)	0 (0.0)	0 (0.0)	0 (0.0)	0 (0.0)	0 (0.0)	0 (0.0)
Pre-term birth & low birth weight	0 (0.0)	0 (0.0)	5 (2.3)	0 (0.0)	0 (0.0)	0 (0.0)	0 (0.0)	0 (0.0)	0 (0.0)	0 (0.0)
IUGR and low birth weight	0 (0.0)	0 (0.0)	1 (0.5)	0 (0.0)	0 (0.0)	0 (0.0)	0 (0.0)	0 (0.0)	0 (0.0)	0 (0.0)
Pre-term birth and fetal death	0 (0.0)	0 (0.0)	1 (0.5)	0 (0.0)	0 (0.0)	0 (0.0)	0 (0.0)	0 (0.0)	0 (0.0)	0 (0.0)
Pre-term birth and fetal distress	0 (0.0)	0 (0.0)	1 (0.5)	0 (0.0)	0 (0.0)	0 (0.0)	0 (0.0)	0 (0.0)	0 (0.0)	0 (0.0)

MHC = Model Health Centre.

Table 4 presented association between maternal complications and prevalence of IDA of the respondents. There is a significant association between maternal complications and IDA (having a *P*-value of .000). Table 5 establishes association between fetal complications and the prevalence of IDA. In this study, there was a significant association between fetal complications and the prevalence of IDA (having a *P*-value of .000).

**Table 4:** Association between maternal complication and the prevalence of iron deficiency anemia of the respondents.

	Iron deficiency anemia and normal MCV classification			^χ2^	*P*-value
	Iron deficiency anemia	Normocytic anemia	Total		
Maternal complications	*F* (%)	*F* (%)	*F* (%)		
Pre-term labor	174 (7.6)	28 (1.2)	202 (8.9)		
No complication	276 (12.1)	765 (33.6)	1041 (45.8)		
Pre-eclampsia	302 (13.3)	26 (1.1)	328 (14.4)		
Post-partum Haemorrhage	327 (14.4)	32 (1.4)	359 (15.8)		
Maternal distress	231 (10.2)	12 (0.5)	243 (10.7)		
Pre-term birth	32 (1.4)	3 (0.1)	35 (1.5)		
Post-natal infection	29 (1.3)	1 (0.0)	30 (1.3)		
Pre-natal infection	2 (0.1)	0 (0.0)	2 (0.1)		
Pre-term birth & post-partum hemorrhage	4 (0.2)	0 (0.0)	4 (0.2)		
Post-partum hemorrhage & maternal distress	8 (0.4)	0 (0.0)	8 (0.4)		
Pre-term birth and maternal distress	4 (0.2)	0 (0.0)	4 (0.2)		
Pre-eclampsia & pre-term birth	1 (0.0)	0 (0.0)	1 (0.0)	1023.650	.000
Pre-term birth and post-natal infection	1 (0.0)	0 (0.0)	1 (0.0)		
Pre-eclampsia and post-natal infection	4 (0.2)	0 (0.0)	4 (0.2)		
Pre-eclampsia and post-natal haemorrhage	5 (0.2)	0 (0.0)	5 (0.2)		
Pre-eclampsia & maternal distress	5 (0.2)	0 (0.0)	5 (0.2)		
Post-natal infection and maternal distress	1 (0.0)	0 (0.0)	1 (0.0)		
Post-partum haemorrhage and post-natal infection	2 (0.1)	0 (0.0)	2 (0.1)		
Total	1408 (61.9)	867 (38.1)	2275 (100.0)		

MCV = mean corpuscular volume.

**Table 5:** Association between fetal complication and prevalence of iron deficiency anemia of the respondents.

	Iron deficiency anemia and normal MCV classification			^χ2^	*P*-value
	Iron deficiency anemia	Normocytic anemia	Total		
Fetal complications	*F* (%)	*F* (%)	*F* (%)		
Low birth weight	350 (15.4)	78 (3.4)	428 (18.8)		
No fetal complications	105 (4.6)	567 (24.9)	672 (29.5)		
Pre-term birth	540 (23.7)	196 (8.6)	736 (32.4)		
Fetal distress	332 (14.6)	23 (1.0)	355 (15.6)		
Fetal death	40 (1.8)	0 (0.0)	40 (1.8)		
Intrauterine growth restriction	24 (1.1)	3 (0.1)	27 (1.2)		
Intrauterine growth restriction & fetal distress	5 (0.2)	0 (0.0)	5 (0.2)	916.753	.000
Fetal distress & low birth weight	4 (0.2)	0 (0.0)	4 (0.2)		
Pre-term birth & low birth weight	5 (0.2)	0 (0.0)	5 (0.2)		
Intrauterine growth restriction & low birth weight	1 (0.0)	0 (0.0)	1 (0.0)		
Pre-term birth and fetal death	1 (0.0)	0 (0.0)	1 (0.0)		
Pre-term birth & fetal distress	1 (0.0)	0 (0.0)	1 (0.0)		
Total	1408 (61.9)	867 (38.1)	2275 (100.0)		

MCV = mean corpuscular volume.

## 4. Discussion

### 4.1. Prevalence of IDA in pregnancy

This study findings showed high prevalence of IDA in pregnancy among respondent with values ranged between 54.1% and 74.0% while the total prevalence across 5 years, the period under investigation was 62.0% at CMHC and RMHC in Obio-Akpor, Rivers State, Nigeria. The alarming increase in IDA prevalence was supported by WHO^[21]^ with 36.5% global prevalence of anemia in pregnant women. In 2021, WHO stated that 50% of anemia in pregnancy was due to iron deficiency but used anemia in pregnancy as an indicator reported that anemia is prominent in India with 88% of pregnant women affected by the condition. In Africa, about 50% of pregnant women are anemic. West Africa is the most affected, and Southern Africa the least. In 2023, 37% of pregnant women are anemic and the largest causes was dietary iron deficiency worldwide.^[2]^ In India, prevalence rates of IDA in pregnant women was 50% to 70%^[10]^ and 64.7% in Uttar Pradesh, India^[20]^ in support of this study findings. Also, similar study conducted in Egypt by Youssry et al^[22]^ supported the finding of this study with 92.8% prevalence of IDA in pregnancy in Egypt using Hb value and serum ferritin from hospital records.

Furthermore, in Lagos and Kano states, Nigeria, prevalence of IDA among pregnant woman was 41%^[13]^ and 40% prevalence of anemia in pregnant women in Latin America and Caribbean by WHO^[21]^ which partially supported the findings of this study. In Akwa Ibom, South Nigeria, rate of occurrence was 61%^[16]^ while Rivers State Port Harcourt, Ringshaw et al^[17]^ reported 60% prevalence of IDA in pregnancy which is in accordance with the findings of this study.

In contrast, the above findings were rejected by Nwanguma et al^[14]^ who reported prevalence of IDA was 23.6% among pregnant women in Enugu State, Nigeria which totally reject this study findings. In rural areas of Rivers State, Nigeria, the prevalence of IDA among pregnant women ranges from 25% to 45.6% in a systematic review by Ugwu and Uneke^[23]^ rejecting this study findings.

### 4.2. Maternal complications associated with IDA in pregnancy

In both health centers, the study major findings were 9.1% to 22.3% of pregnant mother with IDA had pre-eclampsia, 10.5% to 25.1% had post-partum hemorrhage, 7.4% to 22.3% experienced pre-term labor while 14.8% to 27% had maternal distress and 1.3% to 6.4% pre-term birth, and 0.4% had pre-natal infection, while 1.8% to 7.4% had post-natal infection. A related study conducted in Egypt by Youssry et al^[22]^ reported incidence of infection was 2.0% to 31.0% as maternal complications associated with IDA to support above findings while incidence of post-partum hemorrhage was 2.9% to 5.4% which is not in line with above findings. Detlefs et al^[24]^ reported pre-term birth were 5.1% to 8.3% and pre-eclampsia5.9% to 8.3% which partially supported the above findings.

### 4.3. Fetal complications of IDA in pregnancy

In both health centers, the major fetal complications of IDA revealed by this study findings were, 5.3% to 26.6% of pregnant mothers who registered for antenatal from 2019 to 2023 had low birth weight, 11.9% to 48% experienced pre-term, 13.2% to 21.8% had fetal distress, 0.4% to 5.5% experienced intrauterine growth restriction and 2.2% to 6.1% had fetal death. A study on prevalence of IDA and associated complications by Youssry et al^[22]^ reported low birth weight babies was 7.4% to 11.3% and prematurity had 9.0% to 12.9% which were in support of the above findings. Furthermore, Chen et al^[25]^ reported on maternal anemia and neonatal outcome that low birth weight was 1.61% to 6.11% and small for gestational age was 1.37% to 2.61% which were in partial support with study findings. Pre-term were 1.37% to 4.03%, rejecting the study findings.

## 5. Conclusion

IDA in pregnancy is very common among pregnant women in Obio-Akpor, Rivers State, Nigeria, according to this study. The results show that a significant percentage of pregnant women have low Hb levels and low mean corpuscular volume ranging from 54.1% to 74.0% which poses major health concerns to both the mother and the fetus as demonstrated by complications linked to IDA in pregnancy. Targeted public health interventions, such as better nutritional education, iron supplementation programs, and expanded pre-natal care services, are desperately needed to reduce the high prevalence of IDA during pregnancy. Maternal and newborn outcomes can also be greatly enhanced by addressing socio-economic causes and guaranteeing early screening and adequate therapy of IDA during pregnancy. To better understand the long-term implications of IDA during pregnancy and assess the efficacy of management efforts in the area, longitudinal studies are advised.

## Author contributions

**Conceptualization:** Getrude Uzoma Obeagu.

**Data curation:** Getrude Uzoma Obeagu.

**Formal analysis:** Getrude Uzoma Obeagu.

**Investigation:** Getrude Uzoma Obeagu.

**Methodology:** Getrude Uzoma Obeagu, Basil Omieibi Altraide, Emmanuel Ifeanyi Obeagu.

**Project administration:** Getrude Uzoma Obeagu.

**Resources:** Getrude Uzoma Obeagu.

**Supervision:** Getrude Uzoma Obeagu, Basil Omieibi Altraide.

**Validation:** Getrude Uzoma Obeagu, Basil Omieibi Altraide, Emmanuel Ifeanyi Obeagu.

**Visualization:** Getrude Uzoma Obeagu, Basil Omieibi Altraide, Emmanuel Ifeanyi Obeagu.

**Writing – original draft:** Getrude Uzoma Obeagu, Basil Omieibi Altraide, Emmanuel Ifeanyi Obeagu.

**Writing – review & editing:** Getrude Uzoma Obeagu, Basil Omieibi Altraide, Emmanuel Ifeanyi Obeagu.
